# Behavioral pattern separation is associated with neural and electrodermal correlates of context-dependent fear conditioning

**DOI:** 10.1038/s41598-023-31504-z

**Published:** 2023-04-05

**Authors:** Marie K. Neudert, Axel Schäfer, Raphaela I. Zehtner, Susanne Fricke, Rosa J. Seinsche, Onno Kruse, Rudolf Stark, Andrea Hermann

**Affiliations:** 1 Department of Psychotherapy and Systems Neuroscience, Justus Liebig University Giessen, Giessen, Germany; 2 Bender Institute of Neuroimaging, Justus Liebig University Giessen, Giessen, Germany; 3 Center for Mind, Brain and Behavior, Phillips University Marburg and Justus Liebig University Giessen, Giessen, Germany

**Keywords:** Human behaviour, Neuroscience

## Abstract

Hippocampus-dependent pattern separation is considered as a relevant factor for context discrimination and might therefore impact the contextual modulation of conditioned fear. However, the association between pattern separation and context-dependent fear conditioning has not been investigated so far. In the current study, 72 healthy female students completed the Mnemonic Similarity Task, a measure of behavioral pattern separation, in addition to a context-dependent fear conditioning paradigm during functional magnetic resonance imaging. The paradigm included fear acquisition in context A and extinction training in context B on a first day, as well as retrieval testing of the fear and extinction memories in the safe context B (extinction recall) and a novel context C (fear renewal) one day later. Main outcome measures comprised skin conductance responses (SCRs) and blood oxygen level-dependent responses in brain regions of the fear and extinction circuit. Regarding retrieval testing, pattern separation did not correlate with extinction recall, but with stronger dorsal anterior cingulate cortex activation and conditioned SCRs (trend) during fear renewal, indicating a stronger retrieval of the fear memory trace. Our findings suggest that behavioral pattern separation ability seems to be important for context-dependent fear modulation, which is impaired in patients with posttraumatic stress disorder.

## Introduction

The occurrence of conditioned fear in safe contexts is a main characteristic of anxiety disorders and posttraumatic stress disorder (PTSD)^1, 2^. Conditioned stimuli can activate a fear memory trace that elicits a fear response even in safe contexts because of deficits in using contextual information for fear modulation^3–5^. The context-dependent modulation of conditioned fear is therefore of clinical relevance and can be studied with context-dependent fear conditioning paradigms^3, 6–8^: A fear memory trace is acquired during fear learning in context A. During extinction training in context B, an extinction memory trace is formed that inhibits, but does not erase the fear memory trace^10, 11^. After extinction learning, retrieval of the competing memory traces can be tested in different contexts. Retrieval testing in the safe extinction context (‘extinction recall’) leads to a stronger activation of the extinction memory^9^, while the fear memory is activated more strongly during the presence of conditioned cues in the acquisition context or a novel and potentially dangerous context (‘fear renewal’)^7, 10^. The hippocampus is important for context-dependent retrieval of fear and extinction memories^7, 11–13^ by modulating the fear response via direct and indirect projections through the dorsal anterior cingulate cortex (dACC) and the ventromedial prefrontal cortex (vmPFC) to the amygdala^3^.

For adaptive context-dependent responding, contexts must be checked for (dis)similarity to already stored contexts, in order to prepare the organism for a fear response in the presence of conditioned cues in novel, potentially dangerous contexts (e.g., novel context C) or to relax in the case of safe contexts (e.g., extinction context B)^5^. Pattern separation is discussed to be involved in context discrimination^14^ and in memory formation and retrieval processes by storing the diversity of experiences^15^. It extracts the dissimilarity of an input stimulus (e.g., today’s parking lot in a parking garage) that shares similarity to already stored stimuli (e.g., different parking lot yesterday in the same parking garage)^16^. Thus, pattern separation is responsible for encoding a stimulus or event as a dissimilar, non-overlapping memory representation despite its similarity to other stimuli or events (e.g., enables to find the right parking lot each day)^16^. Behavioral pattern separation performance can be measured with the Mnemonic Similarity Task (MST)^17, 18^ and is linked to activity in the dentate gyrus (DG) and CA3 (cornu ammonis) regions of the hippocampus^19, 20^. More recent findings show evidence for the exclusive role of the DG in pattern separation^21^.

Following the neural model of classically conditioned fear generalization in humans^22, 23^, pattern separation processes in the hippocampus should activate structures of the fear- and extinction network including the vmPFC, the dACC, the insula and the amygdala in the case of similarity of a new stimulus/situation with already stored stimuli/situations. For example, if a novel context (e.g., context B or C) shares or includes features of a dangerous context (e.g., context A), pattern separation should initiate and prepare the individual for a fear response by activating the dACC, insula and amygdala, brain regions that are involved in fear excitation and processing^22, 23^, but have different functions. Neurons of the amygdala are involved in the encoding and retrieval of fear memories^3^. The amygdala is considered as a gate keeper, which detects and sends information about emotional stimuli into different processing channels^24^. The insula provides information how salient stimuli affect the body state and is important for interoceptive processes^25, 26^. The dACC is a key region for fear expression^9^ and also involved in the storage of contextual memories^27, 28^. Pattern separation should moreover activate the ventromedial prefrontal cortex, a region known for fear inhibition^29^, in the presence of conditioned safety signals^22, 23^.

Given its role in the integration and differentiation of old and new events^16, 30^, pattern separation may therefore be involved in the context-dependent retrieval of fear and extinction memories. Contextual modulation of conditioned fear requires the discrimination of different, more or less similar contexts in the presence of identical/similar conditioned or generalization stimuli. However, to date, there are no studies investigating the association between behavorial pattern separation and the context-dependent retrieval of fear and extinction memories. Previous studies focused on the association between behavioral pattern separation performance and stronger fear learning and generalization over stimuli^31–33^. Reduced pattern separation performance was associated with a less steep fear generalization gradient and with lower fear inhibition in frontal brain regions and stronger threat expectancies to stimuli that were similar to the conditioned cue^31, 32^, but not with differential fear learning in general^33^.

Expanding previous research, we investigated the association between behavorial pattern separation performance and the context-dependent retrieval of fear and extinction memories in a sample of 72 healthy female participants. The MST^17^ was conducted for assessing behavioral pattern separation performance. Furthermore, participants completed a 2-day context-dependent differential fear conditioning paradigm (adapted from previous studies^2, 6, 7, 13^) during functional magnetic resonance imaging (fMRI). The paradigm included fear acquisition in context A and extinction training in context B on a first day, as well as retrieval testing in context B (extinction recall) and in a novel context C (fear renewal) approximately 24 h later. Neural activation in regions of the fear and extinction network and skin conductance responses (SCRs) were used as dependent variables.

Pattern separation –relevant for processing the (dis)similarity between contexts– is hypothesized to contribute to a better contextual modulation of conditioned fear. During extinction learning, higher pattern separation performance should be associated with a stronger activation increase in the hippocampus and in the vmPFC as well as with an activation decrease in fear-related structures (amygdala, insula, dACC) and with a reduction of conditioned SCRs. With regard to retrieval testing, we further assume that higher pattern separation performance is associated with stronger activation of the hippocampus and with reduced conditioned fear expression during early extinction recall (context B) and stronger conditioned fear expression during early fear renewal (context C). A stronger conditioned fear response should be reflected in enhanced conditioned SCRs and increased activation of fear expressing regions (amygdala, insula, dACC) as well as reduced activation of the vmPFC.

## Methods

### Study procedure

This study belongs to a larger project that investigates the prediction of analog intrusions using the trauma film paradigm based on pattern separation, emotion regulation and context-dependent fear conditioning processes. The whole project consisted of six study days within four weeks and contained clinical interviews, different experimental paradigms and a neuropsychological test battery. For this study, only the relevant paradigms are described below, which were the MST^17^ for assessing behavioral pattern separation (study day 2), the Block-Tapping-Test^34^ as a measure of visuo-spatial memory span from the neuropsychological test battery (study day 2), and the context-dependent fear conditioning paradigm (study days 4 and 5) to assess the context-dependent learning and retrieval of fear and extinction memories. All study procedures were in accordance with the Declaration of Helsinki and approved by the local ethical review board of the Faculty of Psychology and Sports Science at the Justus Liebig University Giessen, Germany.

### Sample

Ninety-four healthy female students recruited via mailing lists at the local university were eligible to participate in this study. All interested students underwent a screening for study in- and exclusion criteria (study day 1), including the Diagnostic Interview for Mental Disorders for DSM-5 (DIPS)^35, 36^ and the Life-Event-Checklist in combination with the Clinician-Administered PTSD Scale for DSM-5 (CAPS-5)^37^. Participants were excluded if they fulfilled the diagnosis of a current or past DSM-5 disorder according to the DIPS/CAPS or reported the experience of a traumatic event within the last four weeks. Participants had to be female and had to report no personal experiences with physical or sexual violence, as we used film scenes during the trauma film paradigm, showing e.g., how a female person was raped by a male perpetrator. Further study exclusion criteria were current and/or regular drug use in the past, color blindness, left-handedness, fMRI contraindications, current or past psychotherapeutic or psychiatric treatment, or medical treatment due to an acute or chronic physical disease.

An a priori power analysis resulted in a minimum sample size of N = 67 for a linear regression analysis with one predictor (Power (1 − β) = 0.80, α = 0.05) with an expected medium effect of ρ = 0.3. Compensating for a drop-out rate of 20% and further drop-outs in main outcome measures (e.g., SCRs), the final sample consisted of N = 94 participants. Twenty-two participants had to be discarded from analyses for the following reasons: early study termination (*n* = 5), technical problems (*n* = 9) or excessive head movements during scanning (*n* = 5) (see below), neurological abnormalities (*n* = 1), technical problems (*n* = 1) or an outlier value in the MST (*n* = 1), leaving a final sample of 72 participants (age: *M* = 23.00 yrs, *SD* = 2.51 yrs, range: 18–31yrs) for analyses regarding the association between pattern separation and context-dependent fear conditioning. The Beck Depression Inventory-II (BDI-II)^38^ as a measure of depressive symptoms and the Symptoms Checklist-90-R (SCL-90-R)^39^ with the Global Severity Index (GSI) as a measure of mental stress indicated low mean scores (BDI-II: *M* = 2.50, *SD* = 3.57, range:0–15; GSI: *M* = 0.15, *SD* = 0.16, range: 0–0.74).

### Mnemonic similarity task

For measuring behavioral pattern separation performance, all participants conducted the MST for objects^17^ (picture set 1) and for scenes^18^ (picture set C), always starting with the MST for objects. The results for the MST for scenes are not reported here. The MST for objects^17^ consists of two phases, the encoding and the following test phase. During the encoding phase, participants viewed 128 pictures of objects for 2 s on a white background presented on a monitor of a laptop (15.6 inches). Participants should indicate via button press on the keyboard if the object is an ‘indoor’ or ‘outdoor’ item (a note card was given as a reminder for the answering options). If they did not give an answer during picture presentation, a white background was presented to avoid differences in picture presentation times within the sample. The next picture was presented on the screen with an inter-stimulus-interval (ISI) of 500 ms, after an answer (forced choice, no time-limitation) was given by button press (no missing responses possible). Immediately after the encoding phase, participants were told that the following test phase is a memory test for the items they had just seen before. During the test phase, 64 pictures of objects presented during the encoding phase (condition: ‘old’), 64 pictures of new objects (condition: ‘foils’), and 64 pictures showing similar but not identical items they had seen during the encoding phase (condition: ‘lure’), were presented for 2 s in a randomized order. For each picture, participants should decide if the presented picture is an ‘old’, a ‘new’ or a ‘similar’ item. After picture presentation, a white background appeared until an answer (forced choice, no time-limitation) was given by button press (no missing responses possible).

The overview of responses for each stimulus and response type of the MST for objects is presented in Table 1. The response distribution of the MST for objects is similar to previous findings in young and healthy samples^31, 40^. Behavioral pattern separation performance was calculated by the ‘Lure Discrimination Index (LDI)’. It is composed of the number of ‘similar’ responses given to lure items (correctly classified lure objects) minus the number of ‘similar’ responses given to foils to correct for a general bias to respond with ‘similar’^41^. Higher behavioral pattern separation performance, as measured with the LDI, represents a better identification of similar items. As described above, two participants were excluded from analyses due to technical problems during the conduction of the MST (*n* = 1) or an outlier value in the number of correctly classified lure items (score > or < 3 × interquartile range, *n* = 1).

**Table 1:** Responses (mean score (maximum = 64) and standard deviations) for each stimulus and response type of the Mnemonic Similarity Task for objects (*n* = 72).

	Old	Lure	Foil
Response: Old	55.31 (4.81)	24.00 (8.47)	1.10 (1.49)
Response: Similar	6.72 (4.11)	34.19 (9.87)	7.31 (4.97)
Response: New	1.96 (2.72)	5.81 (4.72)	55.60 (5.17)

### Block-tapping-test (BTT)

After the conduction of both MST on study day 2, a neuropsychological test battery (not reported here) including the BTT^34^ was carried out. The BTT was used as a covariate in correlational analyses between behavioral pattern separation performance and context-dependent fear conditioning, in order to control for visuo-spatial working memory performance. There was no significant correlation between pattern separation performance and the sum score of the BTT (*r* = 0.175*, p* = 0.143).

### Context-dependent fear conditioning paradigm

#### Experimental design

A 2-day context-dependent differential fear conditioning paradigm was adapted from previous studies^7, 13, 42, 43^. It consisted of different experimental phases: a fear acquisition phase in context A and an extinction training phase in context B on a first day, as well as a retrieval testing phase in context B (extinction recall) and in a novel context C (fear renewal) one day later.

Pictures of different rooms (office room, conference room, and a room with a shelf) served as contexts A, B and C. Each room included the same initially turned off desk lamp. The lamplight colors were used as conditioned stimuli (CS), electrical stimulation as the unconditioned stimulus (UCS). The intensity of the UCS was adjusted in advance to a level, which participants perceived as unpleasant but not painful. The assignment of contexts to experimental phases and the assignment of lamplight colors to CS type condition (CS + /CS −) was counterbalanced and pseudorandomized across participants.

Each trial of each experimental phase started with the presentation of a white fixation cross on a black background (jittered between 0.625 and 2.500 s). Afterwards, a picture of a room with a turned off desk lamp (= context) appeared for 3 s. After three seconds, the desk lamp lit up either blue or yellow for a duration of 6 s. During the fear acquisition phase, one lamplight color (CS +) was followed by an electrical stimulation (UCS) with a duration of 500 ms in 62.5% of the trials; the other lamplight color (CS −) was never followed by the UCS, representing a safety stimulus that should elicit an omission response. In the other experimental phases, the UCS was not administered after CS + or CS − presentation. After CS offset, a white fixation cross on a black background appeared up to a total trial duration of 20 s.

The fear acquisition phase consisted of 8 trials, the extinction training phase of 16 trials, and the extinction recall and the fear renewal phase (retrieval phase) each of 8 trials for each CS type, respectively. Trials were arranged in blocks. Each experimental phase consisted of two blocks. Each block comprised half of the CS trials and contained the same number of CS + and CS − trials. The first two and last two trials of each phase consisted of one CS + and one CS − , respectively. The other CSs were presented in a pseudorandomized order (no more than 2 CS + /CS − in succession). The first CS + was always reinforced during fear acquisition to enhance fear learning. In addition, the last CS + was reinforced to avoid premature extinction learning during fear acquisition.

Participants were informed about the trial structure and content. Before each experimental phase, they were instructed to attentively watch the presentation. They were also informed about the possibility of receiving electrical stimulation after the desk lamp lit up either blue or yellow. They were further instructed that there is a relationship between lamplight color and electrical stimulation. After the fear acquisition phase, participants were asked and informed about the relationship between lamplight color and electrical stimulation, in order to make participants aware of CS-UCS contingencies. We provided information about of CS-UCS contingencies (one lamplight color (CS +) was sometimes followed by electrical stimulation; the other lamplight color (CS −) was never followed by stimulation), to avoid differences in fear and extinction-related processes due to awareness issues^44–47^. Before the extinction training phase started, participants received the instruction that the relationship between light color and electrical stimulation will remain stable over the experiment. This information was given to avoid the idea that CS-UCS contingencies might change over the course of the experiment. It was explicitly stated that the lamplight color that was not followed by stimulation will always be safe and will never be followed by stimulation. The other light color may or may not be followed by electrical stimulation.

#### FMRI data acquisition and analyses

A 3-Tesla whole-body tomograph (Siemens Prisma) with a 64-channel standard head coil was used for image acquisition. We used a T2*-weighted gradient echo-planar imaging sequence (EPI) with 42 slices covering the whole brain (slice thickness = 3 mm; 0.75 mm gap; descending slice order; TE = 30 ms; TR = 2.5 s; flip angle = 81°; field of view = 220 × 220 mm; matrix size = 110 × 110; PAT mode GRAPPA, acceleration factor PE 2) for functional image acquisition (152 volumes for fear acquisition, 280 volumes for extinction training as well as 280 volumes for extinction recall and fear renewal). The first three volumes were excluded from analyses because the magnetization was not yet completely stable. T1-weighted sagittal images were registered (MPRAGE: 0.94 mm slice thickness) and used for normalization procedure. Neuroimaging data were analyzed with Statistical Parametric Mapping (SPM12, r7219, Wellcome Department of Cognitive Neurology, London, UK) implemented in Matlab (R2019a; Mathworks Inc., Sherborn, MA, USA). Preprocessing of the data consisted of the following steps: unwarping and realignment (b-Spline interpolation), slice time correction, co-registration of functional data to each participant’s anatomical image, segmentation of the anatomical image into different tissue types and normalization to the standard space of the Montreal Neurological Institute (MNI) brain. For data smoothing, an isotropic three-dimensional Gaussian filter with a full-width at half maximum (FWHM) of 6 mm was used. Participants (*n* = 5) who showed a framewise displacement^48^ of more than 0.5 mm in at least 15% of the volumes of one scanning session (fear acquisition, extinction training, retrieval testing) were excluded from data analyses.

After preprocessing, first level models were generated separately for fear acquisition, extinction training and retrieval testing (extinction recall and fear renewal). The first level model for the fear acquisition phase contained the regressors context alone, blocks of eight trials for CS + and CS − and blocks of UCS and UCS omission-trials (noUCS-trials). For extinction learning, the regressors context alone and blocks of eight trials of CS + and CS − for early and for late extinction training were included in the model. The first level model for the retrieval testing phase consisted of the regressor context alone as well as of blocks of four trials of CS + and CS − as regressors for early and late extinction recall and for the early and late renewal phase. All first level models also included six movement parameters from the realignment step in addition to one regressor for each volume with a framewise displacement > 0.5 mm^48^. All regressors of interest were modelled based on a stick function convolved with the canonical hemodynamic response function in the general linear model, without specifically modeling the durations of the different events (i.e., event-related design). In addition, we used a high-pass filter of 128 s for filtering voxel-based time series. Autocorrelation of errors was controlled by an AR(1) process. Contrasts between the CS + and CS − were calculated on an individual level for fear acquisition (comparison of the eight CS + and CS − trials), for extinction training (activation decrease from early to late extinction: comparison of the first 8 CS minus last 8 CS trials), as well as for early extinction recall and early fear renewal (comparison of the first four CS + and CS − trials).

The main effects of the context-dependent fear conditioning paradigm are reported in detail elsewhere. In short, neural and SCR data indicated successful fear acquisition in context A. SCRs also indicated successful extinction learning in context B (decrease in SCRs from early to late extinction training). Neural and SCR data showed a return of fear not only during fear renewal in context C (which was expected), but also during extinction recall in context B, when context B should be used as a safety signal. A stronger fear response during extinction recall in the whole sample might be a result of immediate extinction training after fear acquisition^49^. Nevertheless, it still permitted to investigate our research question if differences in pattern separation performance are associated with indices of context-dependent fear conditioning processes.

For analyzing the association between behavioral pattern separation performance and neural correlates of context-dependent fear conditioning processes, multiple regression analyses during second-level analyses implemented in SPM12 were conducted. In a further step, to control for visuo-spatial working memory performance, the multiple regression analyses were repeated adding the sum score of the BTT as a covariate of no interest in the model. Amygdala, dACC, insula, vmPFC and hippocampus, regions of the fear and extinction network^3, 24, 50^, were defined as regions of interests (ROIs). For amygdala, insula, and hippocampus maximum probability masks were taken from the current “Harvard–Oxford Cortical and Subcortical Structural Atlases” provided by the Harvard Center for Morphometric Analysis (http://www.cma.mgh.harvard.edu/) with a probability threshold of 0.50 included in the FSL software package (http://www.fmrib.ox.ac.uk/fsl/). Masks for the left and right dACC and vmPFC were constructed with the MARINA software package^51^ and were used in previous studies^43, 52^. The vmPFC mask consisted of the bilateral medial orbital area of the frontal cortex and the gyrus rectus according to the parcellation of Tzourio-Mazoyer^53^. The dACC mask was defined as the anterior and mid cingulate cortex from the AAL parcellation^53^ in the range of y =  − 17 mm to y = 32 mm, z >  = 12 mm (MNI coordinates). For ROI analyses, the significance threshold was set to α ≤ 0.05 on voxel-level, corrected for multiple testing within each ROI (family-wise error (FWE) correction; using the small volume correction option of SPM12; intensity threshold: *p* ≤ 0.05 uncorrected). Each ROI was tested bilaterally, as we had no hypotheses about laterality. Results with a trend towards significance are reported up to *p* ≤ 0.10. For exploratory whole brain analyses, the significance threshold was set to *α* ≤ 0.05 on voxel-level corrected for multiple testing for the whole brain (FWE-correction); the minimal cluster size (k) for exploratory whole brain analyses was 10 voxels.

#### Skin conductance responses and analyses

SCRs were recorded with a sampling rate of 1000 Hz during all experimental phases using Ag/AgCl electrodes filled with isotonic (0.05 M NaCl) electrolyte medium. The electrodes were positioned hypothenar at the left hand. We used Ledalab 3.4.4 written in MATLAB for preprocessing and data analyses. In a first step, data was downsampled with a sampling rate of 10 Hz and smoothed with a 32 mm Gaussian kernel. A manual data check was performed to check data quality. If technical artifacts were detected in the data, the affected data section was corrected by manual interpolation in Ledalab.

Data were analyzed via ‘trough-to-peak’-analyses^54^. The entire interval response (EIR) was defined as the largest difference between a minimum within a defined time window and the directly following maximum. For CS responses, the time window was set to 0.8–6 s after CS onset, while for UCS responses it was set to 0.8–2.5 s after UCS onset. Contrasts between CS + and CS − (conditioned response) were calculated in parallel with the fMRI analyses. From the final sample of N = 72 participants, data of *n* = 15 participants had to be discarded from SCR analyses because of non-responding during fear acquisition (less than two SCRs > 0.02 μs in reaction to the UCS after CS + (*n* = 12)), technical problems during measurement (*n* = 1), or missing data (*n* = 2), leaving a final sample of *n* = 57 for SCR analyses. In order to examine the relationship between context-dependent fear conditioning processes and behavioral pattern separation performance, correlational analyses according to Spearman’s rho (*r*_s_) were conducted (due to the violation of normal distribution of the SCR data) in IBM SPSS 27 (IBM Corporation, Armonk, USA). We repeated the analyses and added the raw scores of the BTT as a covariate of no interest to the analyses to control for visuo-spatial memory performance.

#### Post-hoc ratings and analyses

After the context-dependent fear conditioning paradigm, post-hoc ratings were conducted outside the scanner to measure context recognition and UCS expectancy for the extinction and novel context. Participants had to decide which of the 6 presented rooms (3 unknown rooms) were used during the paradigm. They should further indicate which room(s) they saw ‘only yesterday’ (≙ the acquisition context), ‘today and yesterday’ (≙ extinction context), or ‘only today’ (≙ the novel context C) and were further asked in which room(s) they had ever received an electrical stimulation after CS offset. For measuring context recognition, a recognition score (overall sum score for correctly classified context assignments) was calculated. After the memory test, participants saw the pictures of the rooms (context A, B, C) which were used during the experimental phases. Valence, arousal and fear were rated on a 9-point Likert scale for each room when the desk lamp was turned off (context alone; no CS condition) or shining blue or yellow (CS condition) (not reported here). To examine UCS expectancy in the different contexts, they also rated how certain they had been that an electrical stimulation immediately appeared after CS offset in the extinction and novel context on a 9-point Likert scale (answer options ranging from ‘sure’ to ‘unsure that the electrical stimulation followed’), respectively. Correlation analyses were conducted to investigate the association between pattern separation performance and post-hoc context recognition and UCS expectancy for the extinction and novel contexts using IBM SPSS 27 (IBM Corporation, Armonk, USA). We repeated the analyses and added the raw scores of the BTT as a covariate of no interest to the analyses to control for visuo-spatial memory performance.

### Consent to participate

All participants spoke German fluently and gave written informed consent. We informed participants that they could terminate the study at any time without negative consequences for them. They were reimbursed for their participation (10 Euro/h or course credits).

## Results

The results of this study show that pattern separation was associated with neural correlates of fear acquisition in context A. Higher pattern separation performance (LDI) was associated with reduced activation in the dACC (T_max_ = 3.99, *p*_fwe_ = 0.046, *k* = 270, MNI: x =  − 2; y = 32; z = 30) and on trend level with reduced activation in the insula (T_max_ = 3.73, *p*_fwe_ = 0.091, *k* = 517, MNI: x = 36; y =  − 12; z = 10) and hippocampus (T_max_ = 3.64, *p*_fwe_ = 0.075, *k* = 172, MNI: x = 34; y =  − 12; z =  − 18) for CS + compared with CS − during fear acquisition in context A. After controlling for visuo-spatial memory performance, the dACC result remained significant (*p*_fwe_ = 0.046), the insula result on trend level (*p*_fwe_ = 0.089), but the hippocampus result did not reach significance any more (*p*_fwe_ = 0.138). In contrast to neural findings, there was no significant association between pattern separation performance and conditioned SCRs during fear acquisition (Spearman's rho (*r*_s_) correlation coefficient: *r*_*s*_ = 0.089, *p* = 0.510). Pattern separation performance was also not correlated with SCRs towards the UCS after CS + compared with the omission response after CS − (*r*_*s*_ = 0.024, *p* = 0.857) during fear acquisition.

**Figure 1:**
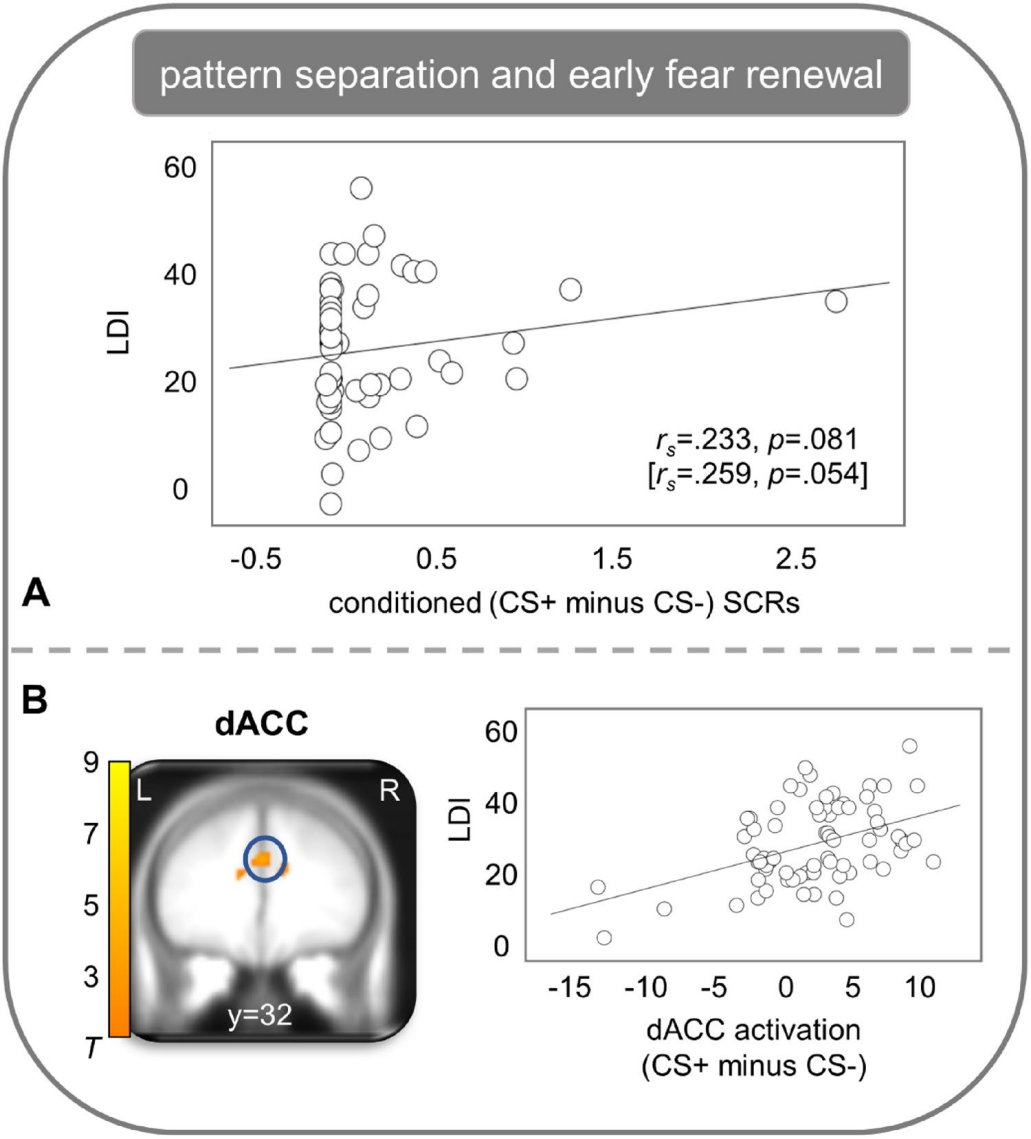
Association between pattern separation performance (LDI: Lure discrimination index) of the Mnemonic Similarity Task for objects and (**A**) conditioned (CS + minus CS −) skin conductance responses (SCRs) using Spearman's rho correlation coefficient (*r*_s_) as well as (**B**) neural activation (CS + minus CS −) in the dorsal anterior cingulate cortex (dACC; contrast estimates of peak voxels) during early fear renewal in context C. Results of the control analyses (controlled for visuo-spatial working memory (sum scores of digit span of the Block-Tapping-Test)) are presented in square brackets. Neural activations were superimposed on the MNI305 T1 template. All coordinates (x, y, z) are given in MNI space. The color bar depicts *T* values. L: left, R: right.

Higher pattern separation performance was not associated with neural (all ROI analyses: *p*_fwe_ > 0.10), but with electrodermal indicators of extinction learning in context B, when the extinction memory trace should be established. Higher pattern separation performance was correlated with a stronger decrease in conditioned SCRs (*r*_*s*_ = 0.333, *p* = 0.011; controlled for visuo-spatial memory performance: *r*_*s*_ = 0.299, *p* = 0.025) from early to late extinction training. Regarding retrieval testing in different contexts after approximately 24 hours, behavioral pattern separation performance was neither correlated with conditioned SCRs (*r*_*s*_ = 0.111, *p* = 0.409) nor neural correlates (all ROI analyses: *p*_fwe_ > 0.10) of extinction recall in context B. During early fear renewal in context C, higher pattern separation performance correlated with stronger conditioned SCRs (trend: *r*_*s*_ = 0.233, *p* = 0.081; controlled for visuo-spatial memory performance: *r*_*s*_ = 0.259, *p* = 0.054) and with stronger activation in the dACC (T_max_ = 4.03, *p*_fwe_ = 0.040, *k* = 694, MNI: x = 0; y = 32; z = 30) and on trend level in the insula (T_max_ = 3.79, *p*_fwe_ = 0.075, *k* = 184, MNI: x = 34; y = 20; z =  − 8,) for CS + compared with CS − (see Fig. 1). DACC results remain significant (*p*_fwe_ = 0.014), but insula results (*p*_fwe_ = 0.150) did not reach significance after controlling for visuo-spatial memory performance. Exploratory whole brain analyses showed no significant associations between pattern separation performance and indicators of context-dependent fear conditioning.

Analyses of post-hoc ratings revealed no association between pattern separation performance and post-hoc UCS expectancy ratings for the safe extinction context (*r* = 0.136, *p* = 0.261), nor for the novel context C (*r* =  − 0.184, *p* = 0.128), also when controlled for visuo-spatial memory performance (all *p* > 0.189). Higher pattern separation performance was associated with a better post-hoc overall context assignment to experimental phases (*r*_*s*_ = 0.352, *p* = 0.003; controlled for visuo-spatial memory performance: *r*_*s*_ = 0.350, *p* = 0.007). However, the results for context assignment are limited in power, since the majority of individuals (*n* = 62) were able to correctly classify all presented context pictures.

## Discussion

The main goal of this study was to investigate the relevance of behavioral pattern separation for context-dependent learning and retrieval of fear and extinction memories. As expected, better behavioral pattern separation performance was associated with electrodermal (SCRs) indicators of stronger extinction learning in context B and electrodermal (trend) and neural indicators of stronger fear renewal in context C, but in contrast to our assumptions not with indicators of stronger extinction recall in context B.

Higher pattern separation performance was related to a stronger decrease of conditioned SCRs from early to late extinction training in context B, when the extinction memory should be acquired. Our results therefore indicate better extinction learning in individuals with higher pattern separation performance. But this interpretation is limited, as neural indicators of extinction learning show no significant association with pattern separation performance. Extinction learning is regarded as a main mechanism for fear reduction during exposure therapy^55^. Pattern separation might be required during extinction learning in context B for encoding the extinction memory as dissimilar from the fear memory trace despite the sharing of identical features. Accordingly, higher pattern separation performance might therefore also explain better post-hoc recognition of the contexts.

In contrast to our expectations, we did not find an association between behavioral pattern separation performance and early extinction recall neither on the neural level nor in conditioned SCRs. It is possible that the retrieval of the extinction memory in a safe context might more likely require pattern recognition, the ability to remember known stimuli, rather than pattern separation which focuses on stimulus dissimilarity^17, 56^. Regarding retrieval testing in the novel context (‘fear renewal’), higher pattern separation performance was associated with stronger conditioned SCRs (trend) and stronger activation in the dACC, a structure relevant for the expression of learned fear^9^. These results might therefore reflect a stronger recall of the fear memory trace in women with higher behavioral pattern separation performance. The return of fear in the presence of conditioned cues in a novel, potentially dangerous context is adaptive by preparing an individual for a potential threat. Impaired pattern separation processes might therefore contribute to difficulties in fear renewal processes which are known for example for patients with PTSD^2^.

Taking a closer look at the neural level, former work on fear renewal emphasizes the interplay between the dACC and the hippocampus. For example, fear renewal was associated with dACC and hippocampus activation and stronger structural connectivity between these regions^7, 57^. In general, the dACC sends input to subregions of the hippocampus which regulate the retrieval of contextual fear memories^28^ and in turn receives input from subregions of the hippocampus important for initiating the fear response^3^. Contrary to our hypotheses, pattern separation was not significantly correlated with activation of the hippocampus during fear renewal. This is in line with findings, showing no association between behavioral pattern separation abilities and hippocampus activation during fear generalization^31^, although the differentiation of stimuli that resemble the CS + are considered to require pattern separation by the hippocampus which then initiates activation of fear- and extinction-related brain regions^22, 23, 58^. The missing hippocampus activation in association with pattern separation in our and previous studies might be related to methodological issues, as e.g., limited resolution of imaging data, preventing the investigation of hippocampal subregions (e.g., dentate gyrus) especially relevant for pattern separation^21^ as well as for encoding and retrieval of memories^59^.

Given the findings of our study, pattern separation may particularly play an important role in the processing of novel stimuli. Context B during extinction learning as well as context C during fear renewal represented unknown contexts for the participants. New contexts need to be checked for (dis)similarities to other already stored contexts, which should require pattern separation. In line with that idea, novelty processing is impaired in Alzheimer's disease^60^, which on the other hand is associated with reduced pattern separation performance^61^. Kesner and colleagues^62^ also reported the relevance of the DG for novelty detection. From mice studies it is known that suppression or lesions of the DG impaired contextual fear extinction^59, 63, 64^ and that its inactivation reduced context-dependent fear renewal^65^. Although we did not investigate neural activation specifically in the DG region of the hippocampus and did not find significant results for the entire hippocampus region of interest, our study also emphasizes the relevance of behavioral pattern separation ability for context-dependent extinction learning and early fear renewal.

The findings of the current study also showed that pattern separation was associated with neural correlates (dACC, insula (trend)) of fear acquisition in context A, when the fear memory trace should be acquired. During the fear acquisition phase, pattern separation might be less required, because context A cannot be compared to a previous context. This could lead to reduced activation in both structures, which are considered to be involved in the retrieval of contextual fear memories and recognition memory^28, 66^, in persons with higher pattern separation performance. These neural findings could also point to less fear expression during fear acquisition, as the dACC and the insula are also fear-related brain regions^9, 24^, to be associated with pattern separation performance. However, the results cannot be interpreted unambiguously and should be treated with caution, because the insula results were only significant on trend level and because there were no correlations with conditioned SCRs. A previous study using SCRs as the main outcome measure for fear learning did also not find an association between pattern separation performance and conditioned SCRs during fear acquisition^33^. However, this was the first study investigating the association between pattern separation and context-dependent learning and retrieval of fear and extinction memories in a female sample which makes comparability to previous findings difficult.

There are further study limitations, which might affect the results. The current findings were based on a young and healthy female sample and cannot be generalized to older individuals, others genders or clinical populations. Additionally, the results should be treated with caution since we did not control for hormonal status and menstrual cycle, which are known to affect emotional learning processes^67, 68^.

## Conclusions

In conclusion, our study shows first evidence for a link between behavioral pattern separation performance and context-dependent learning and retrieval of fear and extinction memories. The findings particularly highlight the relevance of pattern separation performance and underlying neural and electrodermal correlates for early fear renewal. Our data are mainly in line with previous assumptions which consider reduced pattern separation performance as a relevant factor for reduced context discrimination^14^. The current findings further support the idea that maladaptive pattern separation may contribute to deficits in the contextual modulation of fear and the inflexibility of adaptive fear up- and down-regulation known for anxiety disorders and PTSD^2, 5^. However, in order to close the gap between pattern separation, fear conditioning and psychopathology, future studies should investigate the association between pattern separation performance and context-dependent fear conditioning in clinical samples.

## Additional information

We thank our student assistants A. Küss, G. Schneider, S. Koch, D. Meschke, J. Theis, S. Streckmann, K. Ulrich, and M. D’Aloia for help in participant recruitment and data collection. In addition, we thank Dr. C. R. Blecker for technical support. MR-imaging for this study was performed at the Bender Institute of Neuroimaging (BION) at the Justus Liebig University Giessen, Germany.

## Data Availability

The data of this study are available on request from the authors and are not publicly available, as the study also comprises very sensitive clinical information.
